# An Update on Novel Pharmacotherapies for the Treatment of Neuroendocrine Tumors

**DOI:** 10.3390/ijms262211095

**Published:** 2025-11-16

**Authors:** Khalil Choucair, Roupen Odabashian, Sushmita Nanja Reddy, Asfar Sohail Azmi, Muhammad Wasif Saif

**Affiliations:** Barbara Ann Karmanos Cancer Institute, Wayne State University School of Medicine, 4100 John R Street, Detroit, MI 48201, USA; choucairk@karmanos.org (K.C.); odabashianr@karmanos.org (R.O.); azmia@karmanos.org (A.S.A.)

**Keywords:** carcinoid, chemotherapy, immunotherapy, molecular targeted therapies, neuroendocrine tumors, personalized oncology, radionucleotide, somatostatin analogs

## Abstract

Neuroendocrine tumors (NETs) are heterogeneous neoplasms with different molecular characteristics and prognosis. Although slow-growing, NETs are often diagnosed at an advanced stage. The treatment choice depends on primary site, extent, grade, growth rate, somatostatin receptor status, functional status, performance status, and comorbidities. Precise knowledge of the biological and molecular features of NETs has led to the development of novel therapies. Therapeutic options include somatostatin analogs, multi-targeted tyrosine kinase inhibitors (e.g., sunitinib), or mammalian targets of rapamycin (mTOR) inhibitors (e.g., everolimus), telotristat ethyl, chemotherapy, and peptide-receptor radionuclide therapy. Pivotal studies that led to approval, treatment-related adverse events, and safety concerns, as demonstrated in clinical trials and real-world clinical practice. Questions, such as the optimal timing, selection, and sequence of therapies, and biomarkers that predict response to the novel agents in an individual patient, remain to be answered. We propose a stepwise approach for the management of advanced Gastro-entero-pancreatic (GEP)-NETs that utilizes a multidisciplinary team of experts. Biomarkers may assist in both the diagnosis and post-treatment follow-up in patients with GEP-NETs. The next decade of research on GEP-NETs is promising and should provide new insights into the molecular underpinnings of this disease, therapy selection, and the sequencing of the available therapies, along with the potential role of AL in NET pharmacotherapy.

## 1. Introduction

Neuroendocrine tumors (NETs) are a diverse group of neoplasms that can arise from various endocrine glands and produce hormones. They are considered rare, but their incidence has been increasing over time [1]. According to the most recent analysis of data from the National Cancer Institute’s Surveillance, Epidemiology, and End Results (SEER) program, between 2000 and 2020, the age-adjusted incident rate of overall NET in adults was 9.39 per 100,000 [2]. The specific causes for this rise in diagnosis rates are not fully understood, but improvements in imaging technology and better recognition of NET histology are thought to contribute significantly.

In 2000, the WHO established the term NET over the previously used term “carcinoid tumor” in its classification system [3]. These tumors present with diverse clinical manifestations and varying incidence rates across different populations and anatomical sites [4]. The most common primary tumor sites vary by race, with the lungs being the most common in white patients and the rectum in Asian/Pacific Islander, American Indian/Alaskan Native, and African American patients [5]. Within the gastrointestinal tract, NETs of the small intestine are the most frequent, followed by pancreas [1].

Given the highly variable biological features, clinical course, and prognosis, the most recent WHO 2019 classification has established the importance of classifying NETs by primary site, morphological differentiation, and grading [6]. Accordingly, gastro-entero-pancreatic (GEP) NETs [GEP-NETs] are graded into three different categories (G1, G2, and G3) based on the Ki-67 proliferation index, with the G3 category characterized by a Ki-67 > 20% and a well-differentiated morphology across all GEP-NETs.

While the cornerstone of treatment in well-differentiated NETs has traditionally been surgical ablation, or platinum-based for high-grade NETs, recent decades have witnessed the evolution of nonsurgical, systemic therapies that have widened the therapeutic options for these neoplasms. In the review, we will specifically focus on these novel systemic pharmacotherapies.

## 2. Somatostatin Receptor Signaling Pathway and Associated Therapies

### 2.1. Somatostatin Analogs (SSAs)

Somatostatin, a member of the neuropeptide family, is secreted by neuroendocrine, inflammatory, and immune cells in reaction to diverse stimuli [7]. Somatostatin inhibits endocrine and exocrine secretion (such as pituitary GH, TSH, ACTH, gastrin, glucagon, insulin, cholecystokinin, secretin, and vasoactive intestinal polypeptide) and growth factors (including IGF-I, epidermal growth factor, fibroblast growth factor, and vascular endothelial growth factor (VEGF)) [8]. The net effect is reduced movement in the stomach and intestines, decreased gallbladder contraction, and restricted angiogenesis and cell growth. Additionally, SSAs promote cell apoptosis [8].

Its effects are facilitated through a series of G-protein-coupled receptors, known as somatostatin receptors or ssts (sst1–sst5), which are extensively found throughout the brain and body. These five receptors have a high affinity for natural peptides, although only sst2, sst5, and sst3 can bind the shorter synthetic analogs that are employed in the treatment of acromegaly and neuroendocrine tumors [9]. The inhibitory effects of somatostatin are facilitated through its specific interaction with sst2, sst5, and sst1 receptors [8]. These five receptors utilize shared signaling mechanisms, including the suppression of adenylyl cyclase activity, the stimulation of phospho-tyrosine phosphatase (PTP), and the regulation of mitogen-activated protein kinase (MAPK) pathways, all mediated through G-protein-dependent processes [7]. 

Over 75% of GEP-NETs exhibit a high presence of somatostatin receptors, which makes somatostatin analogs particularly suitable for diagnosis and treatment [10]. There is a higher expression of sst2 and sst5 receptors, and the concurrent presence of both correlates with a more favorable prognosis. Conversely, sst1, sst3, and sst4 receptors show weaker expression [10,11,12].

The brief plasma half-life of somatostatin (approximately 1.5 min) prompted the development of more stable synthetic analogs, such as octreotide and lanreotide, which have a half-life of about 2 h. These analogs are utilized in the treatment of pituitary tumors and NETs due to their high affinity for sst2 and sst5 receptors and lower affinity for sst3 receptors, with no binding to sst1 or sst4 receptors. Pasireotide, a newer analog with a plasma half-life of 12 h, is regarded as a universal somatostatin analog because it binds with high affinity to sst1, sst2, sst3, and sst5 receptors [13].

The efficacy of octreotide long-acting release (LAR) in controlling tumor growth and impacting overall survival (OS) was evaluated in the PROMID trial, a placebo-controlled, double-blind, prospective, randomized study of octreotide (30 mg) involving 85 treatment-naïve patients with metastatic NET of the midgut [14]. The median OS was not different between the octreotide (84.7 months) and placebo (83.7 months) groups (HR = 0.83; *p* = 0.51), but subgroup analysis revealed a significant difference based on hepatic tumor load, with a median OS of 107.6 months in the low-tumor-load group compared to 57.5 months in the high-tumor-load group (HR = 2.49; *p* = 0.002). Of note, 88.4% of patients who progressed in the study eventually received octreotide LAR (investigator’s discretion). The PROMID trial highlights that the extent of tumor burden is a critical predictor of survival and that, despite similar OS between the octreotide and placebo groups at randomization, the crossover of placebo patients to octreotide LAR may have influenced the survival data. Side effects related to SSA are predominantly gastrointestinal, commonly including diarrhea or constipation, abdominal pain, nausea, flatulence, cholelithiasis, and gall bladder sludge [15]. SSA can be administered subcutaneously (lanreotide and octreotide) or intramuscularly (depot formulation of octreotide). The recommended starting dose of lanreotide is 60–120 mg administered every 28 days. Patients with NETs are typically treated with octreotide every week initially and then moved to a long-acting (LAR) formulation at a dose of 20 mg every 4 weeks. Currently, the maximum FDA-approved dosage and frequency of LAR is 30 mg every 4 weeks, although several studies within the published literature have described the use of above-label doses for symptom and tumor control of NETs [15].

Lanreotide is another SSA evaluated in the CLARINET study, a randomized, double-blind, placebo-controlled, multinational trial [16]. The study involved patients with advanced well-differentiated or moderately differentiated nonfunctioning SSTR+ NETs of grade 1 or 2. Patients were administered 120 mg of lanreotide (depot) or placebo every 28 days for 96 weeks. The trial showed a significant improvement in PFS for the lanreotide group, with a median not reached compared to 18.0 months for the placebo group (*p* < 0.001), and this benefit was consistent across predefined subgroups. At 24 months, the PFS rate was 65.1% in the lanreotide group versus 33.0% in the placebo group. No significant differences in OS or quality of life were noted between the groups. The most common treatment-related adverse event was diarrhea, occurring in 26% of patients in the lanreotide group and 9% in the placebo group. This study underscores the role of lanreotide in prolonging PFS in patients with metastatic GEP-NET. CLARINE-OLE (NCT00842348) is the open-label extension study of CLARINET, which included 89 patients (lanreotide, n = 42; placebo n = 47) [16]. The median exposure to lanreotide was 59 months, with an overall lower incidence of adverse events compared to the core study and a median PFS of 38.5 months. This study extension thus provided evidence regarding the safety and sustained long-term anti-tumor effects of lanreotide.

While PROMID and CLARINET have established the anti-proliferative effect of SSAs, some differences must be noted: First, PROMID included treatment-naïve patients. In contrast, CLARINET included those with prior treatment exposure. Second, in terms of study subjects, patients enrolled in PROMID had grade 1 NETs and a shorter median time since diagnosis (4.3 months) in comparison to CLARINET (grade 1 and 2, and median time since diagnosis of 33 months). Lastly, patients enrolled in CLARINET had no carcinoid syndrome (exclusion criteria) and had achieved a stable disease (96%) before enrollment in comparison to PROMID (39% had carcinoid syndrome, and all were treatment-naïve). The fact that patients in the CLARINET trial enrolled with a stable disease may explain the fewer tumor-related events, and the differences between the two trials in general, have translated into slight differences in terms of FDA approvals: Octreotide was approved in 1998 for the treatment of severe diarrhea and flushing episodes associated with metastatic carcinoid tumors, while lanreotide was approved in 2007 for the treatment of patients with unresectable, well or moderately differentiated, locally advanced, or metastatic GEP-NET [17,18]. Other differences pertain to the route of administration, dosing, frequency, and therapeutic impact, with lanreotide achieving PFS advantage regardless of disease burden, while octreotide LAR provides OS benefit in low hepatic disease burden [14,15,16,17,18]. Whether or not these two agents can be interchangeable at progression was evaluated by Saif et al. in 91 patients with locally advanced or metastatic GEP-NETs [19]. In this multicenter, non-interventional, retrospective study, patients who had been treated with octreotide for a mean duration of 38.4 months and switched to lanreotide (for at least 90 days) were evaluated. Disease progression was the primary reason for switching (22%), followed by formulary change (15.4%) and patient preference (9.9%). Patients who were switched to lanreotide remained on treatment for a median duration of 24.7 months, with 74% remaining on lanreotide monotherapy by the end of follow-up. Subsequent progression on lanreotide occurred in 24.2% of patients with an overall median PFS on lanreotide, estimated to be 23.7 months (clinician-defined). There was no difference in adverse events during both phases of therapy, indicating no significant differences in safety profiles. This study thus suggested that lanreotide monotherapy could be used as a second-line therapy to stabilize disease and delay progression in patients with locally advanced or metastatic GEP-NETs previously treated with octreotide LAR. Prospective studies are nevertheless warranted to validate this retrospective observational analysis.

The frequency of lanreotide dosing was evaluated in CLARINET FORTE, a phase II trial involving patients with progressive pancreatic or midgut NET [20]. In this trial, 99 patients with grade 1 or 2 midgut NETs or pNETs who experienced disease progression on lanreotide 120 mg every 28 days were treated with the same dose at a shorter frequency of 14 days. Median PFS was 8.3 (midgut) and 5.6 months (pNET), with no deterioration of the quality of life and no treatment-related serious adverse events. The study thus provided grounds for increasing lanreotide dosing frequency in patients who experience disease progression following a standard of care regimen before escalation to less well-tolerated therapies. The question pertaining to the timing of lanreotide therapy (as a second-line after progression vs. maintenance therapy in responders) was addressed in the REMINET trial, a European, multicenter, phase II/III trial, whereby patients with metastatic/non-resectable, well-differentiated GEP-NET patients who had achieved response/stable disease were randomized 1:1 to receive 120 mg of lanreotide or placebo every 28 days [21,22]. At a median follow-up of 27 months, 6-month PFS was 73.1% in patients who received maintenance lanreotide compared to 54.2% for placebo, and median PFS was 19.4 months compared to 7.6 months, respectively. These encouraging results, while not statistically significant, suggest that earlier use of lanreotide as a maintenance therapy in G1 and 2 GEP-NET responders may prolong PFS, and still need to be confirmed.

Pasireotide is a second-generation SSA with high binding affinity to SSTR1, -2, -3, and -5, and a longer half-life, as mentioned above. It is available in a short-acting formulation for subcutaneous injection, which is administered twice daily, and LAR formulation for intramuscular injection, given every 28 days. Pasireotide was initially evaluated in a phase I study involving patients with advanced NETs, which defined 120 mg as the maximum tolerated dose [23]. The study also revealed an encouraging effect on PFS, DCR, and tumor marker (CgA, NSE, IGF-1) reduction. The drug was generally well tolerated, and the most common adverse events (any grade) were fatigue, diarrhea, nausea, and hyperglycemia (79.3% any grade; 10/3% grade 3–4) [23]. A subsequent phase II prospective trial using LAR-pasireotide at a maximum dose of 60 mg in patients with GEP-NET (n = 29) in the first-line setting revealed a median PFS of 11 months, with 4% PR, 60% SD, and 36% progressive disease [24]. Similarly to the prior study, adverse events were mild–moderate, including hyperglycemia (65%) and diarrhea (14%) [24]. LAR pasireotide (60 mg) and octreotide (40 mg) were compared head-to-head in a phase III study involving patients with metastatic NETs and carcinoid symptoms refractory to first-generation SSAs [25]. While the study was halted at an interim analysis (low probability of superiority over octreotide for symptom control), the pasireotide arm had a longer median PFS (11.8 vs. 6.8 months; *p* = 0.045), suggesting the potential antiproliferative activity of pasireotide. While pasireotide is only indicated for certain patients with Cushing’s disease and acromegaly, it remains a promising SSA for NETs, which needs to be explored for additional indication in NET therapy.

The efficacy of LAR-SSA agents at achieving response (PR or CR) in patients with carcinoid syndrome-related symptoms has been recently evaluated and compared in a systematic review and meta-analysis of 17 published trials [26]. With the limitation of significant inter-study heterogeneity (up to 86%), the study revealed a 67% and 68% of pooled CR/PR percentage for diarrhea and flushing, respectively, with no evidence of differential response between the three agents (octreotide, lanreotide, pasireotide) documented in the subgroup’s analyses.

### 2.2. Somatostatin Analog (SSA) Adjunct Therapies

Telotristat ethyl, a newly approved oral inhibitor of tryptophan hydroxylase, has received approval from both the US Food and Drug Administration (FDA) and the European Medicines Agency/European Commission for use in treating diarrhea associated with carcinoid syndrome in adults. This treatment is specifically for patients whose symptoms are not sufficiently controlled by SSA therapy [27,28].

TELESTAR, a crucial phase III randomized trial, established the basis for FDA approval by evaluating the effectiveness of telotristat ethyl in decreasing daily bowel movement (BM) frequency from baseline. The trial defined a positive response as a reduction in BM frequency of at least 30% from baseline for at least 50% of the double-blind treatment period. Notably, 44% of patients receiving 250 mg of telotristat ethyl three times daily (TID) and 42% of those receiving 500 mg TID achieved this response, compared to only 20% of patients in the placebo group [29]. 

In a comprehensive safety assessment from the TELESTAR and TELECAST trials, a considerable proportion of patients administered telotristat ethyl 250 mg (n = 70) and placebo (n = 71) experienced at least one treatment-emergent adverse event (TEAE), with 88.6% and 84.5% of patients reporting TEAEs in the respective groups. Furthermore, 21.4% of patients in the telotristat ethyl group and 16.9% in the placebo group discontinued treatment due to these events. Serious TEAEs were observed during the double-blind phase at rates of 11.4%, 15.7%, and 16.9% among patients receiving telotristat ethyl 250 mg, telotristat ethyl 500 mg, and placebo, respectively. The most frequent adverse reactions (occurring in ≥10% of patients) associated with telotristat ethyl 250 mg three times daily included abdominal pain, elevated γ-glutamyl transferase levels, and fatigue, reported in 26%, 11%, and 10% of patients, respectively. Other common adverse reactions (reported in ≥1% to <10% of patients) were decreased appetite, headache, abdominal distension, constipation, flatulence, increased levels of ALT, AST, and blood alkaline phosphatase, peripheral edema, and pyrexia [30]. Despite its effectiveness in achieving symptomatic control in patients with NETs, the role of telotristat ethyl in disease control and prevention of carcinoid heart disease (valve fibrosis) remains to be investigated. Similarly, three-times-daily dosing remains a challenge in terms of patient convenience and compliance with therapy.

More recently, the TELEPATH phase III, a non-randomized multicenter and open-label trial, evaluated patients who participated in both TELECAST and TELESTAR trials [31]. The study examined the long-term outcome in patients who continued therapy with telotristat ethyl at their specified dose (250 or 500 mg TID) for 84 weeks. A total of 124 patients exposed to telotristat ethyl for a mean of 102.6 weeks were evaluated, and the results support the safety and efficacy of its long-term use, along with an associated sustained improvement in quality-of-life scores (NCT02026063). The TELEACE study is a real-world chart review study that included patients with advanced NETs who received telotristat ethyl for at least 6 months [32]. The study enrolled 200 patients who received telotristat ethyl for an average of 12 months, and revealed a potential antiproliferative effect of therapy along with controlling symptoms of carcinoid syndrome. Telotristat ethyl has also been explored in smaller studies in combination with lutetium peptide-receptor radionuclide therapy (PRRT) in patients with well-differentiated NETs with carcinoid symptoms, and revealed that the addition of telotristat resulted in improved diarrhea, decreased serotonin and flushing (vs. PRRT alone; *p* < 0.004) [33].

### 2.3. Paltusotine

Paltusotine (CRN00808) is identified as a potent, selective, and orally bioavailable non-peptide somatostatin sst2 agonist. It is considered for the potential treatment of acromegaly, neuroendocrine differentiation of prostate cancer, and NETs [34,35].

Paltusotine is available in two formulations: capsules and spray-dried dispersion (SDD) tablets. The capsule formulation, initially used in clinical trials, requires a 2 h post-dose fasting period and has reduced bioavailability when used with proton pump inhibitors, along with non-proportional pharmacokinetics at higher doses. The newer SDD tablet formulation overcomes these limitations by improving solubility and pharmacokinetic performance, offering dose-proportional increases in systemic exposure up to 80 mg, and allowing for greater flexibility in dosing relative to food intake and co-administration with proton-pump inhibitors. This formulation also reduces the necessity for post-dose fasting [36]. Clinical trials are underway to investigate the safety, pharmacokinetics (PK), and exploratory dose response of Paltusotine treatment in individuals with carcinoid syndrome (NCT50361668).

Paltusotine is unique in being the first oral, once-daily, and selective sst2 agonist. Its selectivity against the sst2 receptors, which are expressed in the pituitary somatotrophic cells and the cells of the GEP system, has translated into effective therapeutic benefits for treating acromegaly and GEP-NETs [36,37,38]. Its non-peptide formulation allows for the overcoming of challenges related to peptide formulation such as the inconvenience of scheduling injections, successful delivery of injection (operator-dependent), and the early return of symptoms at the end of the monthly cycle. Additionally, its rapid hormonal suppression makes it attractive for the faster control of symptoms, hence making it ideal for patients presenting with symptomatic GEP-NETs. Preliminary results of a phase II trial were presented recently at the European Neuroendocrine Tumor Society 2024, whereby paltusotine decreased excess bowel movements and flushing by 74% and 72% in a cohort of 16 patients with carcinoid syndrome naïve to SSA or persistent despite SSA, respectively [38].

## 3. Interferon Therapy

The generally low growth rate of NETs reduces the effectiveness of traditional chemotherapy, and a clear extension of survival has yet to be proven [39]. Alpha interferon (α-IFN) demonstrates significant anti-tumor effects in NETs through multiple mechanisms. It exerts anti-proliferative actions by degrading the mRNAs essential for peptide and hormone synthesis, thereby inhibiting protein production. α-IFN also promotes apoptosis and enhances the immune response by increasing the expression of class I antigens on tumor cells, potentially accompanied by the development of autoantibodies [40]. Additionally, α-IFN influences tumor cell differentiation and exhibits cytostatic effects, which help in controlling tumor growth. Furthermore, it normalizes hormone secretion in affected patients, reflecting reduced peptide production or the eradication of malignant cell clones, contributing to its effectiveness in managing these tumors [40,41,42].

Alpha interferon has been evaluated in SSA-refractory cases, in combination with everolimus, as well as fluoropyrimidine (5-fluorouracil) [43,44]. When combined with everolimus, 70% of patients with octreotide-resistant carcinoid symptoms achieved symptomatic improvement, with a mean of 5.1 months duration of symptoms control [43]. The combination of 5-fluorouracil and alpha-interferon led to an ORR in 47% of patients with rapidly progressive NETs (n = 15), with a median response duration of 20.5 months, and 67% symptom improvements [45]. Observational studies have shown that up to 75% of patients experience symptom relief when treated with IFN-α administered subcutaneously at doses of 3–5 million units three times weekly. However, randomized controlled trials have not confirmed these benefits when compared directly with somatostatin analogs alone [46].

The side effects of alpha-interferon treatment, which are dependent on the dosage, primarily consist of flu-like symptoms, fatigue, and minor weight loss. Additionally, approximately 20% of patients experience autoimmune reactions (R30). Due to its significant side effects, IFN-α is typically recommended as a secondary treatment option, after SSA therapy [47].

## 4. Novel Approaches to Targeting NET Growth

Given the heterogeneous clinical behavior, natural history, and prognosis of NETs, advances in molecular techniques, including comparative genomic hybridization, single-nucleotide polymorphism, and, most recently, next-generation sequencing (NGS) technologies, have allowed for a clearer understanding of the biological differences in the landscapes of NETs [48,49,50,51,52,53]. The subsequent discovery of mutations and expression anomalies in specific genes and pathways has set the ground for novel targeted-therapy approaches to targeting NET growth.

### 4.1. Molecular Alterations and Potential Target Pathways

**Figure 1:**
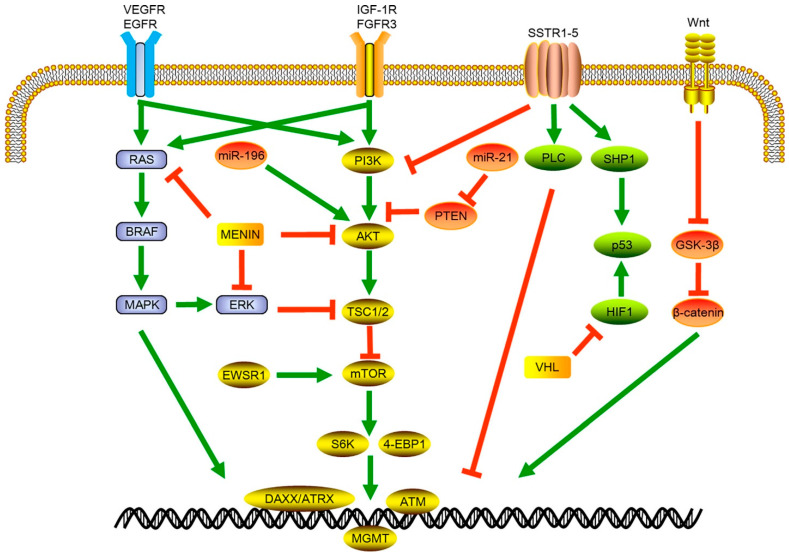
Signaling pathways involved in GEP-NET growth and tumor development. VEGFR: vascular endothelial growth factor receptor; EGFR: epidermal growth factor receptor; BRAF: B-Raf Proto-oncogene; MAPK: mitogen-activated protein kinase; miR: micro RNA; ERK: extra-cellular signal-regulated kinase; EWSR1: EWS RNA binding protein 1; IGF-1R: insulin-like growth factor receptor 1; FGFR3: fibroblast growth factor receptor 3; PI3K: phosphatidylinositol-3-kinase; AKT: protein kinase B: TSC1/2: Tuberous sclerosis 1; mTOR; mammalian target of rapamycin; S6K: 4-EBP1 Eukaryotic translation initiation factor 4E-binding protein 1; DAXX: death domain-associated protein; ATRX: X-linked mental retardation and alpha-thalassemia syndrome protein; ATM: ataxia telangiectasia; SSTR: somatostatin receptor; PTEN: phosphatase and tensin homolog deleted; PLC: phospholipase C; SHP1: Src homology region 2 domain-containing phosphatase-1; HIF1: hypoxia-inducible factor 1; VHL. Von Hippel-Lindau. Adapted from [54].

One of the earliest studies to conduct the full-exome sequencing of well-differentiated GEP-NETs revealed three distinct molecular profiles/pathways enriched with 16 unique gene alterations: the MEN1 pathway, the death-domain associated protein (DAXX)/alpha thalassemia/mental retardation syndrome X-lined (ATRX) pathway (the mammalian target of rapamycin (mTOR)), and the vascular endothelial growth factor (VEGF) pathway [50]. Similarly, whole-genome sequencing of 102 primary GEP-NETs revealed that mutations were most commonly found in the genes involved in four main pathways: chromatin remodeling, DNA damage repair, mTOR signaling, and telomere maintenance [51]. These findings were further confirmed in another study showing a heterogeneous spectrum of mutations and driver events in GEP-NETs that converge into the same four pathways described above [52]. In that same study, however, patients with small-intestine NETs were found to be less dependent on mutational driver events. Instead, tumorigenesis was associated with specific chromosomal alterations (loss of chromosome 8; gains at 4, 5, and 20) and epigenetic events, all converging to hyperactivate the PI3K/mTOR, MAPK, and Wnt pathways [52]. Figure 1 highlights the different pathways involved in NET growth.

#### 4.1.1. MEN1 Pathway

MEN1 alterations mostly pertain to nuclear localization, with around 80% of GEP-NETs cases showing altered Menin localization by immunohistochemistry (IHC), 30% of which were associated with specific MEN1 mutations [55].

#### 4.1.2. The DAXX/ATRX Genes Pathway

DAXX is a histone H3.3 chaperone that binds ATRX, forming a complex that assembles H3.3 nucleosomes, resulting in telomere regulation. Mutations in ATRX or DAXX have been shown to affect telomere regulation, leading to alternative lengthening of telomeres in 61% of GEP-NETs [56]. Further mutational analyses of DAXX/ATRX genes in advanced/developed GEP-NETs revealed that these alterations may be late pathological events, with no DAXX/ATRX mutations or alternative telomere lengthening phenotypes detected in well-differentiated (grade 3, >20 mitosis/HPF) tumors [57]. In an exomic sequences analysis of ten non-familial GEP-NETs, 44% of tumors had somatic, inactivating mutations in MEN1, and 43% had mutations in either of the two subunits of DAXX/ATRX. Clinically, mutations in MEN1 and DAXX/ATRX genes were associated with better prognosis [53].

#### 4.1.3. mTOR Pathway

Alterations in TSC2, PTEN, and PIK3CA genes have been reported in around 14–16% of GEP-NET patients [53,58]. The significance of the mTOR pathway stems from being a master regulator of diverse cellular functions and its subsequent involvement in diverse physiologic and pathologic cellular signaling [59]. PTEN, a negative regulator of mTOR through AKT and TSC2, are downregulated in around 75% of GEP-NETs, with low expression associated with shorter survival [60]. Despite these clear associations between protein expression data and clinical outcomes, newer data from NGS suggest that other molecular mechanisms of mTOR regulation, such as epigenetic changes and post-translational modifications, may be involved in regulating the mTOR pathway in GEP-NETs. Yet, the clinical relevance of this pathway in NET development has been confirmed by the antitumor activity of everolimus, an mTOR inhibitor.

#### 4.1.4. VEGF Pathway

VEGF receptors (VEGFRs) are a family of receptor tyrosine kinases comprising three isoforms, VEGFR1, -2, and -3, with the following distinct ligands: VEGFR1 binds VEGF-A, VEGF-B, and PIGF; VEGFR2 binds VEGF-A and VEGF-C; and VEGFR3 is bound and activated by VEGF-C and D [61]. Activation of the VEGFR pathway leads to the downstream activation of the PI3K/AKT/mTOR pathway and the dysregulation of VEGFR signaling has been demonstrated in NETs. In PanNETs specifically, VEGFR3 activation occurs independently of VEGF ligands and drives the activation of downstream pathways, including mTOR, as well as the ERC pathway, which results in increased vasculogenesis and activation of the epithelial–mesenchymal transition, hence promoting invasion and metastasis [62,63]. In patients with Von Hippel Lindau (VHL), an inherited cancer syndrome in which 17% of patients develop GEP-NET [64], loss of function in the VHL tumor suppressor gene results in constitutive stabilization of HIF1/HIF2-α, which in turn binds and activates the transcription of VEGF genes, hence stimulating angiogenesis [65]. Beyond the VEGF receptors, alternative splicing mechanisms of the insulin-like growth factor receptor (IGFR1) mRNA transcripts have also been described, resulting in around a 2-fold increase in IGF1 stimulation and the activation of the downstream effector pathways described earlier [66].

#### 4.1.5. Other Emerging RTK Signaling in NETs

Despite our better understanding of the molecular signaling underlying NET pathogenesis, everolimus and sunitinib are the only two targeted therapy agents currently in clinical use for patients with advanced diseases. The following other RTKs are being investigated, given their potential as therapeutic targets:

- Epidermal Growth factor receptor (*EGFR*): Dysregulation of EGFR has been implicated in certain types of NETs, namely gastrointestinal carcinoids and GEP-NETs [67]; with increased expression of EGFR, the use of erlotinib in vitro resulted in reduced cell viability and elevated EGFR expression in patients with aggressive PanNETs, correlated with poor prognosis [68]. While infrequent in NETs, *EGFR* mutations and polymorphism have been shown to drive NET differentiation in clinical cases of non-neuroendocrine tumors [69]. Understanding these genetic alterations may help select patients with a predisposition to develop NETs.
- Insulin-like growth factor receptor (*IGF-1R*): IGF type 1 receptor (IGF-1R) is a key player in the pathogenesis of NETs [70,71]. Yet, an exact understanding of this mechanism remains unclear, with a potential dual role in NET biology depending on the tumor stage: a tumor promoter at the early stages, facilitating growth and tumor progression, and a tumor suppressor at the later stages [72]. Understanding this stage-specific distinct tole may allow for the better tailoring of targeted therapies to the specific stage of the disease.
- Rearranged-during-transfection (*RET*) gene: Germline mutations in the RET gene have been identified as the driver alteration in the pathogenesis of medullary thyroid cancer (MTC) [73]. These mutations are constitutively activating, leading to uncontrolled downstream activation of a signaling cascade and net uncontrolled cell proliferation, survival, and tumor progression within the thyroid gland. Among others, the mTOR pathway is activated in these patients with RET mutations [74]. In NETs, including a subset of GEP-NETs, RET mutations or fusions have been implicated in tumorigenesis, including in a case report of successful administration of selpercatinib (a selective RET inhibitor) in a patient with a RET-fusion lung carcinoid tumor [75,76].
- Anaplastic lymphoma kinase (*ALK*): ALK aberrations lead to the constitutive activation of the downstream oncogenic signaling pathways that promote proliferation, survival, and tumor growth [77]. While commonly found and successfully targeted in non-small cell lung cancer, ALK alterations have been recently reported in a subset of NETs, including patients with pulmonary NETs (5.2%), with successful treatment with crizotinib, a selective ALK inhibitor [78,79].

### 4.2. Targeted Molecular Therapies

#### 4.2.1. GEP-NET

The molecular characterizations of the genes and pathways involved in GEP-NET carcinogenesis opened the way for novel research and targeted therapy approaches for those patients. Everolimus, an oral mTOR inhibitor, is approved by the US FDA for the treatment of advanced pancreatic NETs (p-NET) based on results from phase II and multinational double-blind placebo-controlled phase III trials [80,81]. In a phase III study, everolimus significantly prolonged median progression-free survival (PFS) from 4.6 to 11 months compared to placebo (HR: 0.35; 95% CI: 0.27–0.56; *p* < 0.0001) [80]. In patients with functional p-NETs, everolimus also significantly reduces insulin, glucagon, and gastrin secretions, providing a favorable advantage in patients with carcinoid syndrome, as clinically reflected in RADIANT 2 [81,82]. Table 1 summarizes the results of all RADIANT studies involving everolimus.

The high vascularity of NETs gave ground to a strong rationale to target angiogenesis. Sunitinib, a multi-kinase inhibitor of VEGF and platelet-derived growth factor receptor (PDGFR), is now an integral part of treating advanced p-NETs. Sunitinib was initially evaluated in a double-blind placebo-controlled phase III study whereby sunitinib 37.5 mg daily revealed an improved median PFS from 5.5 to 11.4 months (HR: 0.42; 95% CI: 0.26–0.66) [86]. Subsequent studies further consolidated the role of VEGF inhibition in treating p-NET [87,88,89]. The study led to the approval of sunitinib for treating p-NETs, hence changing the previously existing treatment paradigm. It is worth noting that attempts to combine everolimus and sunitinib were severely limited by poor tolerance, hindering the adoption of this combination in routine clinical use [90]. Questions about the most appropriate sequencing of therapy remain to be answered. In a retrospective analysis of 92 patients with well-differentiated NETs of different origins (57 pNETs), patients treated with everolimus first (followed by sunitinib at progression) achieved better disease control rates and median PFS compared to those treated with sunitinib first [91]. This was also observed in patients with pNETs, thus suggesting that everolimus may be preferable as a first therapy compared to sunitinib when targeted molecular therapy is initiated following disease progression.

Other targeted therapies involving pan-RTKs have been evaluated in clinical trials. Surufatinib is a small-molecule TKI that selectively targeted VEGFR-1, -2, and -3, fibroblast growth factor receptor 1 (FGFR1), and colony-stimulating factor 1 receptor (CSF-1R), and has been evaluated in two Chinese phase III trials of patients with pNETs (SANET-p trial) and extra-pancreatic NETs (SANET-ep) [92,93]. In both trials, the drug was evaluated in patients who had progressed on two lines of prior systemic therapies, and, compared to placebo, revealed a PFS benefit, with an overall acceptable safety profile. While approved in China, the FDA did not approve surufatinib indication in the US. Cabozantinib has recently been evaluated in a patient population similar to the SANET trials, and results from the CABINET trials published in September 2024 revealed significant improvement in PFS in previously treated pancreatic and extra-pancreatic NETs compared to placebo [94]. The drug is currently under review by the FDA but has not yet been fully approved for NET therapy.

#### 4.2.2. Non-GEP-NET

Despite lower response rates compared to GEP-NETs, several single-arm phase II studies have supported a role for angiogenesis inhibitors in advanced non-GEP-NETs, with a substantial effect on delaying tumor growth [87,88,89]. In one randomized trial, patients with NETs, including GEP and non-GEP tumors, were randomly assigned to receive bevacizumab or pegylated IFN-α-2b [95]: patients who received bevacizumab had a response rate of 18% and an improved PFS at week 18 (95% vs. 68%). A subsequent pivotal phase III SWOG trial randomized 427 patients to receive long-acting repeatable (LAR) octreotide with either bevacizumab (n = 214) or IFN-α-2b (n = 213) [96]; while no significant differences in PFS were observed between the two arms (15.4 vs. 10.6 months; HR: 0.90; 95% CI: 0.72–1.12; *p* = 0.33), patients who received bevacizumab had higher confirmed response rates of 12% vs. 4% for IFN-α-2b. In the SWOG S0518 trial evaluating the combination of bevacizumab with depot octreotide, bevacizumab was administered intravenously at a dose of 15 mg/kg every 21 days [96]. In terms of treatment-related toxicities, the combination was associated with hypertension (32%), proteinuria (9%), and fatigue (7%), consistent with the known toxicity profiles of either drug.

Similarly to GEP-NETs, everolimus was also evaluated in a phase III trial (RADIANT-2) in patients with progressive, well-differentiated NETs and carcinoid syndrome [81]: four hundred and twenty-nine patients were randomly assigned to receive LAR octreotide with either everolimus or placebo. The combination with everolimus showed a clinically important 5.1 months improvement in PFS (from 11.3 to 16.4 months; HR: 0.77; 95% CI: 0.59–1.00; *p* = 0.026).

#### 4.2.3. Dosing and Toxicity

Of the above-discussed targeted therapies, only everolimus and sunitinib are FDA-approved for clinical use. Everolimus was evaluated in RADIANT-2 (everolimus + depot octreotide) and RADIANT-3 (everolimus monotherapy) trials [80,81]. In both trials, everolimus was administered orally at a dose of 10 mg once daily. Stomatitis (62–64%), rash (37–49%), and diarrhea (27–34%) were the most reported side effects. Patients also experienced fatigue (31%), and infections (23%) with hyperglycemia (5%) and anemia (6%) were more frequently seen in patients treated with everolimus.

In the phase III trial that led to Sunitinib’s approval ([86]/NCT00428597), sunitinib was administered at a dose of 37.5 mg orally daily. The most frequent adverse events (AEs) in the sunitinib group were diarrhea, nausea, vomiting, fatigue, and asthenia. Table 2 summarizes the most common toxicities.

#### 4.2.4. Challenge of Emerging Resistance

Despite increased response and survival rates with the use of sunitinib and everolimus to treat NETs, tumors are likely to develop resistance to therapies. Currently, there is interest in understanding the mechanisms of resistance to these active drugs, including the role played by the tumor microenvironment (TME) and stroma. For everolimus, it is hypothesized that the inhibition of TORC1 results in the upregulation of AKT via increased insulin-like growth factor (IGF)/PI3K-dependent pathway signaling, hence bypassing the inhibition of TORC1 altogether [53,80]. Accordingly, strategies to downregulate IGF expression with somatostatin analogs (octreotide, pasireotide) or directly inhibit IGF signaling using monoclonal antibodies (cixutumumab) are being developed. Other strategies to overcome resistance to everolimus include the dual blockade of PI3K and mTOR and TORC1 and TORC2 using serine-threonine kinase inhibitors such as BEZ235 and INC128, respectively (reviewed [97]). Resistance to antiangiogenic therapy with sunitinib is also a challenge, with preclinical data suggesting a role of the increased expression of transcription factors that control the expression of pro-angiogenic molecules [98,99]. Hence, strategies to target multiple proangiogenic pathways (VEGF, MET, FGF, etc.) using a pan-TKI molecule, are in development. Targeting these transcription factors (e.g., hypoxia-inducible factor, or Sp1) is another strategy to prevent therapeutic resistance to Sunitinib.

### 4.3. Immunotherapy

There are currently no NET-specific indications for immune checkpoint inhibitors (ICIs). Despite the success of ICIs in Merckle cell carcinoma (MCC), a rare NET of the skin, and small-cell lung carcinoma (SCLC), a high-grade NET of the lung, the use of ICIs to treat GEP-NETs is not yet recommended as a preferred regimen. In those tumors, the National Cancer Center guidelines (NCCN v1. 2023) recommend ICIs within tumor-agnostic indications based on specific biomarkers: Microsatellite Instability—High (MSI-H), deficient mismatch repair (dMMR), and tumor mutational burden—high (TMB-H), and only in patients who progressed following prior treatment with no satisfactory alternative treatment options [28].

#### 4.3.1. ICI Monotherapy

When used alone, ICI has shown low response rates in NETs. Pembrolizumab (anti-PD-1) was evaluated in a non-randomized phase Ib trial, KEYNOTE-028, in a large cohort of patients with advanced solid tumor expression, PD-L1 [100]. The cohort contained 15 patients with GEP-NET, of whom 1 (1/15; 6.67%) had an objective response and 14/15 (93.3%) experienced stable disease. Progression-free survival (PFS) rate was 40% at 6 months and 27% at 12 months, and 1-year OS rate was 87%. In a phase II KEYNOTE-158 trial, three patients with p-NET and one with rectal NET achieved a partial response after a median follow-up of 24 months [101]. Subsequent studies evaluated the efficiency of pembrolizumab in patients with or without PD-L1 expression, showing no differences in disease control rate, PFS, or OS based on PD-L1 expression [102]. In an open-label phase II trial, 107 patients with well-differentiated NETs of the lungs, appendix, small intestine, colon, rectum, or pancreas who previously failed standard therapies, were treated with pembrolizumab [101]. The objective response rate (ORR) was 3.7% only, and the median PFS was 4.1 months. Only 4 patients with GEP-NET (40 with p-NET, 25 with small intestine, and 18 with other GI NETs) had a partial response (3 with p-NET, 1 with rectal NET) [101].

Toripalimab (humanized IgG4 anti-PD1 antibody) has also been investigated in patients with recurring or metastatic NETs after first-line therapy. In a phase Ib trial involving 40 patients with NETs, the overall ORR was 20% with a median duration of response of 15.2 months [103]. Of these, the ORRs were 22.2% (2/9) for p-NETs, 13% (3/23) for non-pancreatic GI NETs, and 37.5% (3/8) for non-GI NETs. Interestingly, ORR was 50% in patients with PD-L1 expression ≥ 10%, and 10.7% in those with PD-L1 < 10% (*p* = 0.019).

Last, spartalizumab (humanized anti-PD1 antibody) is another ICI that was evaluated as a monotherapy in a phase II single-arm study of patients with well-differentiated, pretreated metastatic G1/G2 NET (32 GI, extra (e)-p-NET and 33 p-NET) [104]. ORR was 3.1% in GI-NET and 3% in p-NET.

While spartalizumab had limited efficacy in NETs, its AEs were noted to be mild and manageable, with fatigue (29.5%) and nausea (10.5%) being the most frequently reported events. Similarly, the previously mentioned ICI monotherapies had mild and manageable AEs. With pembrolizumab treatment, for example, only 21.5% of treatment-related adverse reactions were grade ≥ 3, with the frequent AEs being malaise (22.4%) and diarrhea (13.1%) [101].

#### 4.3.2. Combination ICI Therapy

The combination of nivolumab and ipilimumab, an antibody that targets the cytotoxic T lymphocyte-associated antigen-4 (CTLA-4), was evaluated in patients with NETs in a phase II clinical trial, DART (NCT02834013). Fifteen patients with extra-pancreatic NET (e-p-NET) received nivolumab/ipilimumab [105]. The objective responses were observed only in high-grade tumors (ORR: 44%), with poor efficacy in the well-differentiated tumors (ORR: 0%; low and intermediate grade), suggesting a benefit from this combination in aggressive, high-grade tumors only. A subsequent phase II trial of the ipilimumab/nivolumab combination involving 29 patients with advanced NETs revealed an overall ORR of 24%, and 43% for p-NETs specifically [106].

#### 4.3.3. ICI and Targeted Anti-Angiogenesis

Basic research has provided evidence for a synergistic effect when combining ICIs and anti-angiogenic therapies [107]. In a phase II basket trial administering atezolizumab (1200 mg) and bevacizumab (15 mg/kg) every 3 weeks in solid tumors, patients with G1/G2 NETs who had progressed on prior therapy were included [108,109]. Data from the GEP-NET (n = 20) and non-GEP-NET (n = 20) cohorts revealed ORRs of 20% and 15% and median PFSs of 14.9 and 14.2 months, respectively. These findings suggest that GEP-NET patients might benefit from this combination.

#### 4.3.4. Chemo-Immunotherapy Combinations

While successful in other solid organs, the combination of ICIs and chemotherapy has yielded unsatisfactory results in patients with GEP-NETs. In a phase II trial including 22 patients with extrapulmonary, poorly differentiated NETs who progressed on prior first-line therapy, the combination of pembrolizumab and chemotherapy (77% treated with irinotecan and 23% with paclitaxel), the ORR was 9%, with partial response achieved only in two patients, and stable disease in 14% of cases [110]. The NICE-NEC study evaluated the combination of chemotherapy plus ICIs in the first-line setting and included 38 patients with G3 NETs [111,112]. Of those, 81.6% were GEP-NETs and 68% were poorly differentiated neuroendocrine carcinomas. The combination revealed a promising therapeutic effect with an ORR of 54.1% and a median PFS of 5.7 months, hence suggesting that adding nivolumab to chemotherapy for G3 NETs has potential effectiveness, without significantly added toxicities.

In summary, current evidence to support the use of ICIs in GEP-NET remains immature, despite preliminary data suggesting potential benefits from a combinatory approach using ICIs, mostly in high-grade, poorly differentiated tumors. The often-low TMB and “cold” immune tumor microenvironment (TME) of NETs further support using combination therapies to overcome NET’s intrinsic resistance to ICIs. This also highlights the need for biomarkers to select patients with GEP-NET who could benefit from ICIs. While MSI-H/dMMR, TMB, and PD-L1 expression are key biomarkers to predict the benefit of ICIs, the incidence of MSI-H/dMMR in NETs is relatively low [113]. TMB presents with its limitations owing to the way TMB is calculated, and the choice of significant cut-off with the current definition of TMB-H (>10 mut/Mb) still unable to predict the benefit of ICIs in all types of cancer, including NETs [114,115]. As discussed earlier, PD-L1 positivity was associated with ORRs in some trials. However, PD-L1 expression appears to be heterogeneous across the different studies, with some studies showing no relevance to ICI efficacy [101,102,116,117]. Exploring the immune TME and the role of immune cell infiltration is a potential avenue that is currently being investigated [118,119,120].

### 4.4. Peptide Receptor Radionuclide Therapy (PRRT)

Peptide-receptor radionuclide therapy (PRRT) in NET has been extensively researched, particularly in somatostatin receptor-positive tumors, since the 1990s. PRRT is a radiolabeled somatostatin analog that specifically targets somatostatin receptors on neuroendocrine cancer cells.

Indium 111-pentetreotide [^111^In-DTPA Octreotide] was one of the first radiolabeled SSAs studied in NET. A study of 27 patients with pancreatic islet cell tumors treated with ^111^In-DTPA Octreotide demonstrated a survival benefit. Despite not showing significant radiological tumor regression, patients treated with PRRT had a median OS of 18 months, which was higher compared to standard therapies involving somatostatin analogs and chemotherapy, which were the standard of care at the time of the study [121].

Other PRRTs like Lu-Dotatate and yttrium-90 have been extensively researched and have shown significant clinical and objective responses. A phase I multicenter study was carried out in veterans to assess the safety of ^90^Y-DOTA^0^, Tyr^3^ Octreotide [^90^Y-DOTATOC] in patients with NET. In this open-label dose-escalating study, patients receiving ^90^Y-DOTATOC were compared to those receiving ^111^In-DTPA Octreotide as the control group. Compared to patients treated with ^111^In-DTPA Octreotide, those who received ^90^Y-DOTATOC had a substantial improvement in OS and PFS. Subsequent larger studies further confirmed the benefits of ^90^Y-DOTATOC in NET patients. The most observed side effects included hematologic, renal, and gastrointestinal toxicities [122].

In patients with GEP-NET specifically, Lutetium Oxodotreotide [^177^Lu-Dotatate; Lutathera^®^] is a radioactive SSA that specifically targets somatostatin receptors on these tumors, delivering radionuclides directly to cancer cells to induce cell death. A pivotal NETTER-1 trial in 2018 was crucial in procuring US FDA approval for PPRT for patients with GEP-NET [123]. In this randomized phase III trial, patients with advanced progressive midgut NETs that were somatostatin receptor-positive were randomized (1:1) to receive ^177^Lu-Dotatate (at a dose of 7.4 GBq every 8 weeks) with octreotide-LAR administered intramuscularly at a dose of 30 mg (n = 229) or octreotide-LAR alone (control) administered intramuscularly at a dose of 60 mg every 4 weeks (control arm). Patients treated with ^177^Lu-Dotatate had significantly longer 20-month PFS of 65.2% (95% CI: 50.0–76.8) compared to 10.8% (95% CI, 3.5 to 23.0) in the control group, with a response rate of 18% vs. 3%, respectively. However, the final OS analysis difference of 11.7 months did not show statistically significant differences among the two groups. Hematologic toxicities were the most common adverse event noted among the treatment group [123].

Similarly, the phase III NETTER-2 study investigated ^177^Lu-Dotatate in patients with advanced grade 2 or 3 well-differentiated GEP-NET. This study reaffirmed the benefits of ^177^Lu-Dotatate in prolonging PFS by 14.3 months (22.8 vs. 8.5 months in treatment vs. control, respectively) and achieving a higher ORR compared to the standard treatment, (43% vs. 9.3%). Besides hematologic toxicities, it is important to be vigilant for the occurrence of myelodysplastic syndrome, as evidenced by a reported case in the group receiving 177Lu-Dotatate. Thus far, Lu-Dotatate has shown to be highly effective in treating patients with NET, NETTER-2 is the first randomized study to establish a first-line treatment option in advanced GEP-NETs [124].

Some major differences between NETTER-1 and NETTER-2 trials should be noted: NETTER-1 focused on grade 1 and 2 NETs of the midgut, while NETTER-2 enrolled grade 2 and 3 GEP-NETs (55% p-NET). NETTER-2 evaluated patients in the first-line setting, compared to NETTER-1 where patients were pretreated, with evidence of progressive disease. The primary endpoint in both studies was PFS; however, the key secondary endpoint in NETTER-1 included ORR and OS, while NETTER-2 only examined ORR and safety outcomes.

Several ongoing trials are exploring the efficacy of PRRT either alone or in combination with other systemic treatments. One such trial is the COMPETE trial, a prospective multicenter open-label phase III study comparing the effectiveness and safety of PRRT with ^177^Lu-Edotreotide to Everolimus in patients with unresectable, progressive, SSTR+ GEP-NETs. This study, which began in February 2017, is set to be completed in December 2024. More recently, the results from ALPHAMEDIX 02, a phase II, open-label, multicenter trial evaluating the safety, tolerability, and efficacy of ^212^Pb-DOTAMTATE in PRRT-naïve (cohort 1) and PRRT-refractory (cohort 2) subjects with unresectable or metastatic SSTR+ GEP-NETs, were presented at the 2024 Annual American Society of Clinical Oncology meeting [125]. The ^212^-Pb-DOTAMTATE was administered at a dose of 67.6 µCi/kg/cycle (maximum activity: 5.5 mCi) every 8 weeks, for up to four cycles. The primary endpoints included ORR and incidence of adverse events, with secondary endpoints including PFS and OS. Only data from cohort 1 (PRRT-naïve) was presented: 17/36 patients with metastatic SSTR+ GEP-NET achieved a confirmed response (ORR: 47.2%; 95% CI: 32–63%). So far, 7/17 patients (41%) with confirmed responses had a duration of response (DOR) ≥ 6 months, with one patient having a DOR ≥ 12 months. In terms of side effects, lymphopenia was the leading cause of the 59% of grade 3–4 events in cohort 1. In total, four fatal adverse events were reported (death/progressive disease, n = 2; carcinoid syndrome, n = 1, and sepsis, n = 1). Despite a small sample size and short follow-up times, treatment of PRRT-naïve advanced/unresectable GEP-NETs with up to four cycles of ^212^-Pb-DOTAMTATE was well tolerated, with side effects similar to ^177^Lu-DOTATATE, but substantially higher ORRs.

### 4.5. Systemic Chemotherapy

Cytotoxic chemotherapy is the standard therapy for patients with poorly differentiated NET. The role of chemotherapy in patients with well-differentiated NET is less defined.

#### 4.5.1. Alkylating Agents

##### Streptozotocin-Based Regimens

Streptozotocin, derived from *Streptomyces achromogenes*, was among the earliest chemotherapies approved by the FDA for the treatment of PanNETs in 1982. This antibiotic alkylates DNA, leading to strand breaks, and apoptosis, ultimately inhibiting DNA replication and cell proliferation. The efficacy of streptozotocin in NETs was first investigated in the 1970s by Broder et al., specifically focusing on pancreatic islet cell tumors. Their study revealed improved OSs and ORRs, with 37% of patients (14/38) showing a measurable disease response > 50% [126].

Streptozotocin was later explored in combination with various systemic chemotherapies such as 5-fluorouracil (5-FU), doxorubicin, and cyclophosphamide. In the 1970s, Moertel et al. conducted a study where 103 patients with unresectable islet cell carcinoma were randomly assigned to receive either streptozotocin alone or in combination with 5-FU. Patients who received the combination had significantly better ORRs compared to streptozotocin alone (63% vs. 36%), with a higher rate of complete response (CR; 33%) in the combination group. While the difference in OS was not statistically significant, it held clinical significance, with patients in the combination arm showing longer survival. Gastrointestinal toxicity, nephrotoxicity, and hepatotoxicity were the most observed adverse events associated with streptozotocin. This study established a new standard of care for treating NET, emphasizing a combination regimen involving streptozotocin and 5-FU [127,128].

##### Dacarbazine-Based Regimens

Dacarbazine, an alkylating agent, has been investigated in the treatment of metastatic glucagonoma since the 1970s, and multiple smaller studies have shown promising results.

In E6282, a phase II trial, dacarbazine (850 mg/m^2^ IV, every 4 weeks) was evaluated in 50 patients with p-NET [129]. Of those patients, 56% were treatment naïve, and the overall ORR was 33%, with a higher ORR in treatment-naïve (50%) compared to pretreated patients (13.6%). The median duration of response was 10 months, and the median OS was 19.3 months. In terms of toxicity, 30% experienced grade 3–4 toxicities, mostly hematologic and gastrointestinal. In another, albeit retrospective, study, 50 patients with p-NETs were treated with dacarbazine (650 mg/m^2^, IV, every 4 weeks), and 32% of patients achieved a partial response, with a median PFS of 10 months [130].

##### Temozolomide-Based Regimens

Temozolomide is an alkylating agent that works by methylating DNA at the O6 position of guanine, inhibiting DNA replication and cellular apoptosis, typically repaired by a protein called methylguanine methyltransferase (MGMT). While traditionally used to treat glioblastoma multiforme, researchers have also investigated temozolomide in combination with capecitabine for NETs.

Capecitabine, on the other hand, is a fluoropyrimidine that is converted into 5-FU, a thymidylate synthase inhibitor, thereby blocking DNA synthesis. It is believed that capecitabine may reduce the levels of MGMT, which could enhance temozolomide’s ability to suppress MGMT activity. Prior smaller studies have reported promising benefits with this combination, which was thus studied further in a larger cohort.

In a multicenter, open-label, phase II trial led by the ECOG-ACRIN cancer research group, 144 patients with advanced low-grade or intermediate-grade p-NET were randomized 1:1 to receive a combination of temozolomide and capecitabine or temozolomide alone. The treatment regimen involved temozolomide 200 mg/m^2^ by mouth once daily on days 10–14 and capecitabine 750 mg/m^2^ by mouth twice a day for days 1–14, repeated every 28 days, or temozolomide 200 mg/m^2^ by mouth once daily on days 1–5, repeated every 28 days alone (control arm). The results showed a significant improvement in PFS in the combination arm (22.7 months vs. 14.4 months, HR: 0.58; *p* = 0.022), with an ORR of 39.7% [131]. Accordingly, temozolomide and capecitabine have been established as a good treatment choice for patients with GEP-NET. Note that for a select group of patients with glucagonoma, systemic chemotherapy could be used, with streptozocin-based regimens and capecitabine–temozolomide combination [132].

Temozolomide (150 mg/m^2^ orally, day 1–7 and day 15–21) was also evaluated in combination with bevacizumab (5 mg/kg, IV day 1 and day 15) in patients with advanced NETs [133]. With ORR as a primary endpoint, 34 patients (44% with p-NET) were treated, and the ORR was 15%, with all responses occurring in patients with p-NET. The median PFS was 11 months for all patients and 14.3 months for those with p-NETs. Toxicities included thrombocytopenia (18%), nausea/fatigue (15%), fatigue (6%), neutropenia (6%), and proteinuria (3%).

Of note, while dacarbazine and temozolomide share the same active metabolite (5-(3-methyltriazen-1-yl) with temozolomide, temozolomide is administered orally and is better tolerated, replacing dacarbazine for patients with NETs [134]. The efficacy of either agent was not compared head-to-head, but a retrospective analysis of 247 patients treated with either dacarbazine/5-FU (84/247) or temozolomide/capecitabine (153/247) revealed no difference in ORRs (38.3% vs. 39.2%; *p* = 0.59) [135].

In glioblastoma, MGMT methylation testing is commonly performed, as this has shown differences in tumor response to temozolomide. However, retrospective data from NETs have shown inconsistent results [136].

##### Oxaliplatin-Based Agents

Oxaliplatin-based combinations with fluorouracil (FOLFOX) or capecitabine (CAPOX) have been the most studied in patients with well-differentiated NETs.

In patients with p-NETs, FOLFOX demonstrated significant antitumor activity: in a retrospective analysis of 31 heavily pretreated PanNET patients, 25 received FOLFOX and 6 received FOLOFOX plus bevacizumab [137]; the ORR was 45% with a median PFS of 6 months and OS of 15 months, with no statistically significant differences between the two regimens. All tumor grades were included, although most patients had grade 2 tumors (51.6% vs. 9.7% grade 1, 19.4% grade 3, and 19.4% unknown). In terms of toxicity, the most common adverse events included neuropathy, hepatic encephalopathy, neutropenia, fatigue, and hypoglycemia. In a modified FOLFOX-6 regimen (oxaliplatin 85 mg/m^2^ IV on day 1, leucovorin 100 mg/m^2^ on day 1, bolus fluorouracil 400 mg/m^2^ IV day 1, and continuous infusion of 5-FU, 2400 mg/m^2^ over 46 h, every 14 days) in 48 patients with advanced well-differentiated NETs (33 with GEP-NETs), ORR was 33%, DCR was 81.8%, median PFS was 12.6 months, and median OS was 29.4 months [138]. In a pooled analysis of two prospective phase II studies of FOLFOX plus bevacizumab (every 14 days) or CAPOX plus bevacizumab, ORR was 41.7% in patients treated with FOLFOX and 18.8% with CAPOX, with median PFSs (for GEP-NET) of 21 and 15.7 months, respectively, and median OSs of 31 and 38 months, respectively [139]. In patients with e-p-NETs (all with grade 2 tumors; n = 155), oxaliplatin-based regimens elicited less tumor cytoreduction with lower ORRs and DCRs [140].

##### Other Chemotherapy Agents

Poorly differentiated p-NETs and e-p-NETs are aggressive types of cancers and, hence, are considered and treated like small-cell lung cancers with a platinum-based chemotherapy regimen, i.e., cisplatin or carboplatin in combination with etoposide (EP).

A recent multicenter phase III randomized controlled trial, ECOG-ACRIN EA2142, was conducted to study if CAPTEM was superior to Cisplatin/EP in grade 3 GEP-NETs. The interim analysis revealed a prolonged PFS of 5.3 months for patients receiving EP compared to 2.43 months for those receiving CAPTEM, along with OS rates of 13.6 months and 12.6 months, respectively [141]. The study was, however, terminated, as it did not demonstrate superiority.

Gemcitabine in combination with capecitabine or temozolomide and irinotecan in combination with 5-FU or cisplatin are some of the other alternative chemotherapeutic drugs that have been less well studied in metastatic NETs and have demonstrated efficacy in controlling tumor progression.

Irinotecan, an inhibitor of DNA topoisomerase 1, was investigated in combination with cisplatin (IP) for treating advanced poorly differentiated GEP-NETs. A phase II randomized trial found that the objective response rate was comparable between IP and EP in small-cell NETs. The study did not show significant differences in PFS or OS [142].

In summary, the role of chemotherapy with well-differentiated NETs remains an area of investigation. While patients with p-NETs appear to be the most chemo-sensitive, those with e-p-NETs derive less benefit from this approach. On the other hand, the platinum-etoposide regimen remains the cornerstone for G3 NET. Similarly, head-to-head comparisons between different regimens across different NET subtypes remain limited, but temozolomide-based and oxaliplatin-based regimens appear to be the most used chemotherapies in advanced settings. The role of chemotherapy in adjuvant and neoadjuvant settings remains undefined. Combinations of chemotherapy with targeted therapies and PRRT may pave the way to more systemic cytoreductive therapies that could be effective across a broader range of NETs.

## 5. Discussion

NETs are a diverse set of tumors and are also genetically varied. Unfortunately, clinical diagnosis is usually delayed in most cases until metastases are present. The presence of somatostatin receptor expression in over 75% of GEP-NETs makes somatostatin analogs particularly suitable for diagnosis and treatment; however, only a few studies have comprehensively investigated the expression of all five of the SSTR subtypes, and thus knowledge regarding the correlation of their expression with clinical outcomes in NETs is incomplete. Patients with unresectable, metastatic GEP-NETs usually undergo systemic therapy aiming at controlling symptoms, limiting tumor growth, and prolonging life. From that perspective, and given the expected long-term treatment, it is crucial to carefully select the appropriate therapeutic strategy that balances achieving the desired outcomes of appropriate symptom control while limiting long-term toxicities to ensure a good quality of life. Factors that need to be accounted for include tumor characteristics (location, grade, and proliferation index) as well patient’s comorbidities and extent of symptoms. We propose a summary of these factors, referred to as the “8 Ss” approach (site, size, stage, sex, SSTR status, secretory status, severity, and score), as summarized in Table 3. Understanding these factors is crucial to making therapeutic decisions regarding the optimal timing, agent selection, and sequence of therapies. There is currently no standardized approach that applies to all patients with GEP-NETs. Rather, we recommend a more personalized approach for the management of GEP-NETs. SSAs are typically the first-line therapy to control the growth of well-differentiated NETs, and, despite slight differences in approved indications, octreotide and lanreotide are used interchangeably in clinical practice. Lutathera/PRRT has revolutionized the treatment for patients who express SSTR on NET cells. The FDA approved Lutathera in January 2018 to treat adults with SSTR-positive GEP-NETs based on the results of the NETTER-1 trial (Grade 1 and 2 midgut NETs). Most recently, the results of the NETTER-2 trial (Grade 2 and 3 GEP-NETs) were published, aiming to move Lutathera to the front-line stage and to grade 3 NETs. Such therapies come with a spectrum of toxicities, and familiarity with hematologic toxicities is of utmost importance.

For tumors that are resistant to SSAs or rapidly progressing, multiple therapeutic options exist, depending on the location of the primary tumor and the histological grade. The available options for pNETs are everolimus, sunitinib, or chemotherapy, usually capecitabine plus temozolomide. For GI-Nets, the possible options are IFN or streptozotocin-based systemic chemotherapy or everolimus. Due to the lack of predictive biomarkers, the selection between different therapeutic options or sequences is usually based on subjective clinical judgment, tumor characteristics (site of the primary, histologic grade, etc.), and toxicity profile of each anticancer agent. For example, a VEGF inhibitor may be most optimal agent to be administered to patients with uncontrolled diabetes mellitus or active lung disease, while an mTOR inhibitor could be the suitable choice in patients with uncontrolled arterial hypertension, cardiac history, or high risk of hemorrhage. Chemotherapy is usually the best option for patients with rapidly progressive disease after failing targeted agents. Since there is no consensus on the optimal sequencing of therapy in GEP-NETs, we recommend a tailored approach to be adopted in each patient based on the features specific to the patient (age, co-morbid conditions), tumor (primary site, burden of disease, grade), and drug (toxicity profile, acceptance by patient) under a multidisciplinary team.

We recommend the follow-up and monitoring of treatment response for patients who undergo resection with curative intent, as well as those with advanced disease, as late recurrence can occur in some patients. While the maximum duration of follow-up is not well defined, we favor an initial follow-up of 3–6 months after resection, and then every 6–12 months thereafter for at least 7 years. In patients with advanced disease, surveillance is recommended every 3–6 months, which can be increased to every 6 months in those with long duration (>12 months of stable disease). Modalities of assessment include blood and urine chromogranin A and 5-HIAA levels (consider testing at baseline, then follow up only if abnormal), anatomic imaging (multiphasic CT or MRI; recommended at baseline and every subsequent visit), and nuclear imaging (octreotide scintigraphy; considered to be clinically indicated and for clinically suspected recurrence).

The heterogeneity of NETs limited the discovery of biomarkers to therapy. Hopefully, predictive biomarkers in the future may lead to tailored therapy according to every patient’s disease characteristics. Similarly, a better understanding of the resistance mechanisms, including the role of the TME, different mutations in the mTOR pathway, and transcriptional regulators of pro-angiogenic molecules, will help sequence therapies and develop individualized treatment-specific resistance/susceptibility biomarkers. To that end, developing biomarkers to predict response and resistance, and for post-treatment follow-up, is warranted. For example, research on biomarkers related to the mTOR pathway will need to combine the genomic alteration of the different effectors, along with the final protein expression and function, to improve the selection of candidate patients to receive mTOR-targeted agents. Expression levels of PTEN and TSC2 have recently been linked to outcomes [58]. Similarly, the response to VEGF inhibitors may be predicted with functional imaging [143]. MicroRNAs (miRNA) are another class of biomarkers recently investigated in NETs, and in PNETs more specifically, and have been shown to potentially predict clinical outcomes [144,145].

In patients treated with temozolomide-based chemotherapy, emerging data suggests that high levels of MGMT expression may drive resistance to temozolomide, potentially providing a biomarker (low MGMT levels) to select patients for treatment [146]. Biomarkers with potential prognostic value could further help in selecting patients for therapy versus active surveillance: elevated plasma levels of chromogranin A (CGA) and neuron-specific enolase (NSE) at baseline were shown to be associated with significantly shorter PFS and OS [147]. Furthermore, early CGA and NSE response were linked to favorable therapeutic benefits, highlighting further potential role in monitoring response (and determining the subsequent frequency of treatment) for patients on active therapy. Lastly, biomarkers for ICIs ought to be better characterized given the relatively poor performance of this class of medications in NETs. Given the limitations and performance variability of ICI biomarkers, there is a need to investigate co-occurring biomarkers that, when coupled with the existing ones, would provide better predictions regarding immunotherapy benefits. More recently, somatic mutation analysis has emerged as a novel approach for biomarker identification via the potential characterization of NET transcriptomic signatures; compared to comprehensive genomic analysis, this approach was shown to provide better sensitivity and specificity to predict the course of NETs [148].

In summary, the incidence of NETs continues to rise, and the disease itself is heterogeneous, coupled with a complex landscape of treatment options without any clear guidelines or biomarkers. Currently, therapeutic decisions around the choice of therapeutic agent or treatment sequence are based on clinical criteria. The discovery of predictive biomarkers will eventually lead to the achievement of more precise treatments.

## 6. Conclusions

**Figure 2:**
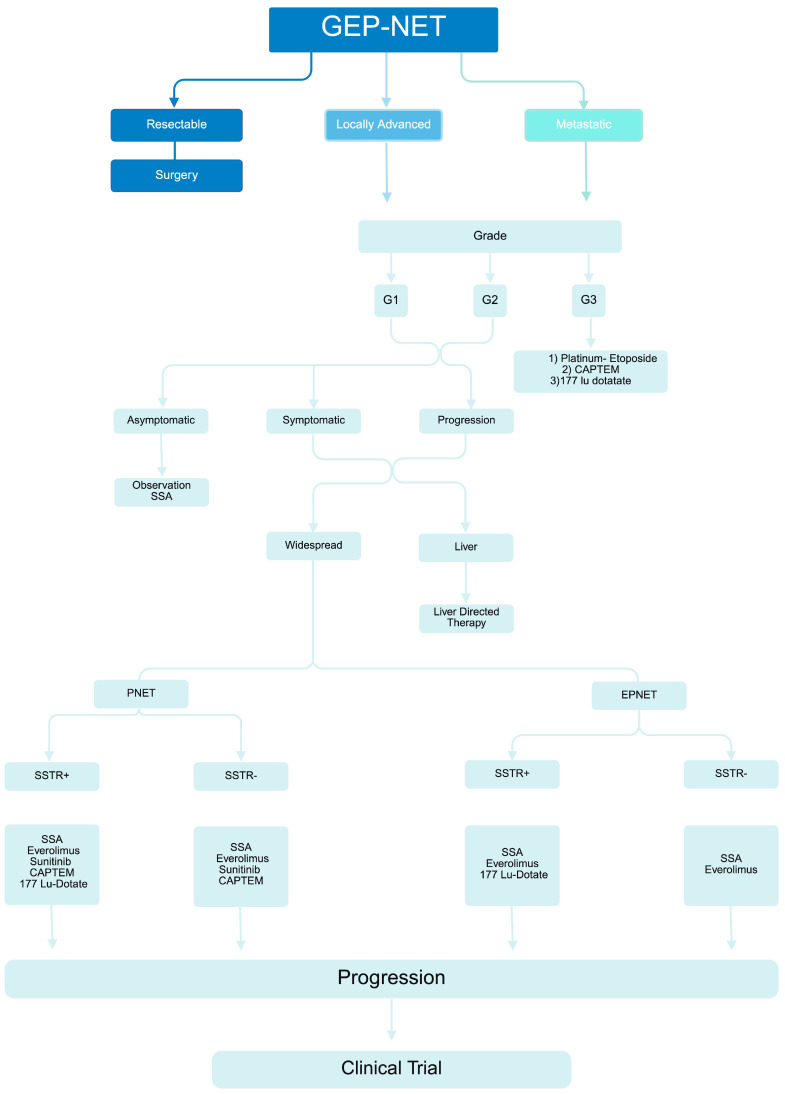
Proposed algorithm for the management of advanced GEP-NET. GEP-NET: gastro-entero-pancreatic neuroendocrine tumor. G1, G2, G3: grade 1, grade 2, grade 3; SSA: somatostatin analog; p-NET; pancreatic NET; e-p-NET: extra-pancreatic NET; SSTR+: somatostatin receptor-positive; SSTR−: somatostatin receptor-negative; CAPTEM: capecitabine-temozolomide.

The marked heterogeneity amongst NETs’ natural history, biological behavior, and prognosis is certainly reflected by the wide array of therapeutic strategies currently available. Proper classification based on location, morphology, degree of differentiation, and proliferation rate, as well as distinguishing functional tumors from non-functional ones, are all essential to tailor therapy through a multidisciplinary team approach. While surgery remains the mainstay of therapy with curative intent for non-metastatic tumors, long-acting SSA is the main treatment for most secretory tumors, with new agents such as telotristat or, more recently, paltusotine now available with better bioavailability and as adjunct therapies for symptomatic control. For patients with advanced disease, molecular advances have opened the way for targeted therapies including TKIs, mTOR inhibitors, and radionuclides therapy. For high-grade poorly differentiated NETs, treatment with chemotherapy (in combination with immune checkpoint inhibitors for SCLC) remains the standard of care, given the exquisite chemosensitivity of the tumors. Immunotherapy is perhaps the most recently explored therapeutic opportunity in NETs, and awaits the development of selective, personalized biomarkers to identify patients who would respond and the development of potential combinations to incite an immune response in these natural “cold” tumors. Figure 2 provides a proposed stepwise algorithmic approach for the management of GEP-NET based on our multidisciplinary approach at Karmanos Cancer Institute.

## Figures and Tables

**Table 1:** Summary of all RADIANT trials, evaluating the use of everolimus in patients with NETs following progression on prior therapies.

TRIAL	Phase	Population	Intervention (I)	Control (C)	Outcomes
RADIANT 1 [83]	2	Adv. pNETs, (n = 160)	Everolimus (10 mg/d) +/− octreotide LAR *	N/A	Stratum 1 (n = 115; no LAR): ORR 9.6%; SD: 67.8%; mPFS: 9.7 mo; Stratum 2 (n = 45; +LAR): ORR: 4.4%; SD: 80%; mPFS: 16.7
RADIANT 2 [81]	3	LGNETs with CS (n = 429)	Everolimus (10 mg/d) + octreotide LAR *	Octreotide LAR	mPFS: 16.4 (I) vs. 11.3 mo (C)
RADIANT 3 [84]	3	Adv. pNETs (n = 410)	Everolimus (10 mg/d)	Placebo	mPFS: 11 (I) vs. 4.6 mo (C) mOS: NR in both groups
RADIANT 4 [85]	3	WD (G1/2) Adv. NETs with no CS ** (n = 302)	Everolimus 10 mg/d	Placebo	mPFS: 11 (I) vs. 3.9 mo (C) DCR: 82.4% (I) vs. 64.9% (C)

Adv.: advanced; pNETs: pancreatic neuroendocrine tumors; LGNETs: low-intermediate grade neuroendocrine tumors; CS: carcinoid syndrome; WD: well-differentiated; G1/2: grade 1 or 2; mg/d: milligrams per day; LAR: long-acting repeatable; N/A: not applicable; ORR: objective response rate; SD: stable disease; mPFS: median progression-free survival; mOS: median overall survival; NR: not reached; mo: months; DCR: disease control rate; * 30 mg every 28 days; ** Gastrointestinal or lung origin.

**Table 2:** Summary of the most common adverse events in FDA-approved targeted molecular therapies. In RADIANT trials, the most common adverse events occurred in >10% of patients; for NCT-00428597, this was defined as >15%. LAR: long-acting, repeatable; NR: not reported.

	RADIANT-2		RADIANT-3		NCT-00428597	
Adverse Event	Everolimus 10 mg Daily (+ LAR Octreotide)		Everolimus, 10 mg, Daily		Sunitinib 35.5 mg Daily	
Grade	All Grades	Grade ≥ 3	All Grades	Grade ≥ 3	All Grades	Grade ≥ 3
Stomatitis	62%	3.7%	64%	14%	22%	4%
Rash	37%	0.9%	49%	1%	18%	0%
Diarrhea	27%	6%	34%	3%	59%	5%
Fatigue	31%	7%	31%	2%	32%	5%
Hyperglycemia	12.1%	5.1%	27%	5%	NR	NR
Anemia	16.3%	1.8%	35%	6%	NR	NR

**Table 3:** Summary of different factors to be considered for therapy selection—the 8 “S” approach. GEP: Gastro-entero-pancreatic; NET: neuroendocrine tumor; p-NET: pancreatic NET; e-p-NET: extra-pancreatic NET; STTR: somatostatin receptor; PRRT: peptide receptor radionuclide therapy; G: grade; CAPTEM: capecitabine/temozolomide.

Characteristic	Details
**S**ite	GEP (including p-NET vs. e-p-NET, and liver-dominant disease) or non-GI NET
**S**ize	Size of ≥20 mm: prognostic for recurrence and metastasis
**S**tage	Resectable vs. locally advanced vs. metastatic
**S**ex (pace of growth/proliferation)	Rate of tumor growth; typically assessed with imaging every 3 months
**S**STR status	For: STTR+ consider PRRT
**S**ecretory (hormone)	Functional/secretory NET: benefit from surgical resection if possible, targeted therapy or PRRT vs. non-functional: can be observed unless advanced (consider chemotherapy)
**S**everity (burden)	Disease burden as a prognostic marker for survival
**S**core (grade)	G3 (vs. G1,2) tumors: consider platinum-etoposide, CAPTEM, and ^177^Lu-Dotatate

## Data Availability

No new data were created or analyzed in this study. Data sharing is not applicable to this article.
